# *Lepidium meyenii* Walpers Promotes the Regeneration of Salivary Gland and Prevents Xerostomia After Irradiation Injury

**DOI:** 10.3390/nu17193033

**Published:** 2025-09-23

**Authors:** Yi-Ting Tsai, Yuan-Chuan Lin, Ming-Jen Cheng, Chun-Ming Shih, Chien-Sung Tsai, Ze-Hao Lai, Ching-Yi Wu, Chen-Wei Liu, Feng-Yen Lin, Yi-Wen Lin

**Affiliations:** 1 Division of Cardiovascular Surgery, Tri-Service General Hospital, National Defense Medical University, Taipei City 11490, Taiwan; 2 Taipei Heart Institute, Taipei Medical University, Taipei City 11031, Taiwan; 3 School of Post-Baccalaureate Chinese Medicine, Tzu Chi University, Hualien City 97004, Taiwan; 4 Department of Life Science, Fu Jen Catholic University, New Taipei City 242062, Taiwan; 5 Department and Graduate Institute of Pharmacology, National Defense Medical University, Taipei City 11490, Taiwan; 6 Institute of Oral Biology, National Yang Ming Chiao Tung University (Yangming Campus), Taipei City 112304, Taiwan; 7 Department of Basic Medical Science, College of Medicine, University of Arizona, Phoenix, AZ 85721, USA; 8 Division of Cardiology and Cardiovascular Research Center, Taipei Medical University Hospital, Taipei City 11031, Taiwan; 9 Departments of Internal Medicine, School of Medicine, College of Medicine, Taipei Medical University, Taipei City 11031, Taiwan

**Keywords:** xerostomia, *Lepidium meyenii*, macaene, macamide

## Abstract

Objectives: *Lepidium meyenii* Walpers (LMW), a high-altitude plant, is known to stimulate hormone release, counteract neurodegeneration, and protect against oxidative stress. Saliva is vital for oral health, and reduced production leads to xerostomia, often caused by aging, radiation, or Sjögren’s syndrome. Key pathological features include mesenchymal fibrosis and acinar atrophy, largely regulated by the TGF-β1 pathway. Current treatments are limited, with many patients relying on artificial saliva. Developing therapies to restore salivary function could offer significant benefits. Methods: In this study, we assessed the protective effects of LMW extract (LMWE) in irradiated C57BL/6J mice and TGF-β1-treated rat parotid acinar cells (Par-C10) using histological, molecular, bioenergetic, and 3D organoid analyses to evaluate salivary gland regeneration and lineage-specific differentiation. Results: LMWE significantly restored gland weight, shortened secretion lag time, and increased amylase activity in irradiated mice. Histological and molecular analyses showed reduced acinar atrophy and fibrosis, preservation of epithelial polarity, and upregulation of Mist1, AQP5, and amylase. In vitro, LMWE protected Par-C10 cells from TGF-β1-induced senescence, preserved mitochondrial membrane potential, and improved epithelial barrier function. In 3D organoid cultures of Par-C10 cells embedded in matrix, (1*E*,4*Z*)-1-(2,4-dihydroxyphenyl)-5-(3,4-dihydroxyphenyl) penta-1,4-dien-3-one (DHPPD) and (*Z*)-*N*-phenyldodec-2-enamide (E4Z-PD)-selectively enhanced acinar and ductal lineage differentiation, respectively. Conclusions: These results suggest that LMWE promotes salivary gland regeneration through antioxidative and lineage-specific mechanisms and may represent a safe and effective therapeutic strategy for xerostomia.

## 1. Introduction

The salivary gland is an exocrine gland composed of acini, duct systems, myoepithelial cells, and mesenchymal cells. Saliva is crucial for oral health and functions, including digestion, speech, taste, chewing, swallowing, antimicrobial action, cleaning, pH buffering, and lubrication [1]. Xerostomia is defined as an altered saliva composition or reduced saliva volume. Xerostomia can occur at any age and may cause health issues such as tooth decay, difficulty swallowing, oral infections, or malnutrition if it persists, significantly impacting the quality of life [2]. Three common causes of xerostomia are: (1) Radiation therapy for head and neck cancer, which can impair acinar cell function, decrease saliva flow, and reduce amylase secretion [3]; (2) Sjögren’s syndrome, an autoimmune disorder in which overactive immune cells infiltrate the salivary gland, leading to cytokine production and tissue proteolysis, causing acinar cell death and impaired secretion [4,5,6]; and (3) aging, during which acinar cells undergo apoptosis and are replaced by fat tissue and extracellular matrix, gradually diminishing saliva secretion capacity [7,8].

Current clinical treatments for xerostomia include the use of artificial saliva to mimic the rheological properties of natural saliva [9]. In addition, mouthwashes or toothpaste can temporarily relieve dry mouth; help maintain oral, dental, and gum health; and enhance pH buffering [10]. However, these methods only provide short-term symptom relief and do not address the underlying causes. For head and neck cancer patients, pilocarpine, a cholinergic parasympathomimetic drug, is administered after radiation therapy to stimulate saliva secretion [11]. However, because the muscarinic acetylcholine receptors of pilocarpine are distributed throughout the body, it often causes significant side effects such as nausea, diarrhea, frequent urination, excessive sweating, vasodilation, bronchoconstriction, and hypotension, limiting its clinical use [12,13]. Amifostine (Ethyol, WR-2721) is the only FDA-approved drug for the prevention of xerostomia [14]. When injected 15–30 min before radiation therapy, alkaline phosphatase in normal cells hydrolyzes WR-2721 into WR-1065, a metabolite with cytoprotective effects against radiation damage [15]. However, amifostine often causes side effects, with over 50% of patients experiencing vomiting and hypotension. The cytoprotective properties of amifostine are mainly attributed to its antioxidant capacity, which scavenges free radicals, donates hydrogen atoms, and releases endogenous thiol groups (such as GSH) to protect cellular proteins [16]. Given the similar metabolic mechanisms in normal and cancerous cells, reducing free radical-induced damage with antioxidants may help alleviate radiation- and chemotherapy-related toxicity [17]. Recent studies have demonstrated that local injection of amifostine and WR-1065 into the submandibular glands of C57BL/6 female mice prior to a single 15.0 Gy radiation dose significantly enhances acinar cell survival and preserves glandular function. Compared to intravenous administration, local ductal injection minimizes systemic side effects, such as hypotension, nausea, vomiting, and allergic reactions [18]. Nevertheless, more than half of patients still experience mild vomiting and hypotension during amifostine treatment. Ongoing research is evaluating its impact on chemotherapy-induced toxicities, including nephrotoxicity, ototoxicity, neurotoxicity, and myelosuppression [19]. Thus, the development of an effective and low-side-effect treatment for xerostomia is urgently needed. Several edible plants and herbal formulations have been investigated for their therapeutic potential in xerostomia, particularly among cancer patients receiving radiotherapy. Systematic reviews and randomized controlled trials have highlighted the benefits of traditional Chinese herbal medicines, including formulas such as Liu-wei-di-huang-wan (Yukmijihwang-tang) and Sha Shen Mai Dong Tang, which are composed of edible and medicinal plants like Ophiopogon japonicus (Mai Dong), Adenophora/Glehnia root (Sha Shen), Rehmannia glutinosa, and Prunus mume (Wu Mei) [20,21,22,23]. These remedies have been shown to improve subjective dryness, enhance salivary flow, and alleviate radiation-induced oral complications. Narrative reviews further support the use of food-medicine homologous plants such as Trichosanthes root (Tian Hua Fen) and Panax notoginseng (San Qi) as adjunctive therapies [24,25]. Collectively, these findings suggest that edible herbal medicines offer promising complementary approaches for managing xerostomia and improving oral health-related quality of life.

*Lepidium meyenii* Walpers (LMW), commonly known as maca or Peruvian ginseng, is a biennial herbaceous plant of the cruciferous family that primarily grows in the highlands of the Peruvian Andes and the Qinghai-Tibetan Plateau in China [26]. As an edible plant, the root of LMW has long been consumed as a traditional food and medicine by the indigenous people of the Andes [27], where it is valued not only for its pharmacological properties but also for its nutritional composition. Maca root is rich in carbohydrates, dietary fiber, essential amino acids, vitamins (such as vitamin C and B-group vitamins), and minerals (including calcium, iron, and zinc), making it a functional food that contributes to both energy supply and micronutrient intake. It is well-adapted to low temperatures, intense UV radiation, and low atmospheric pressure [28]. Studies have shown that regular consumption of LMW can slow the progression of chronic neurodegenerative diseases such as Alzheimer’s, Parkinson’s, and Huntington’s diseases [29]. Animal research has demonstrated that LMW promotes the pituitary secretion of follicle-stimulating hormone and luteinizing hormone in female rats, thereby enhancing fertility [30]. Additionally, LMW increases superoxide dismutase activity and glutathione (GSH) levels in the liver, significantly reducing plasma lipid and cholesterol levels [31]. LMW also extends endurance in load-bearing swim tests and reduces blood urea nitrogen (BUN) and lactate accumulation during exercise [32]. LMW root extract (LMWE) contains several bioactive compounds, including non-starch polysaccharides, polyphenols, glucosinolates, alkaloids, essential amino acids, macaenes, and macamides [33,34]. LMW polysaccharides notably inhibit liver lipid peroxidation caused by carbon tetrachloride [35], while macaenes and macamides enhance male sexual function [34,36]. Moreover, Dini et al. demonstrated strong antioxidant activity of *Lepidium meyenii* through radical scavenging assays, supporting its role in reducing oxidative stress [37]. A subsequent comprehensive review reported that Maca extracts possess anti-inflammatory, neuroprotective, and anti-fibrotic effects in both in vitro and animal studies, mediated via macamides and glucosinolates [38]. Although direct data on TGF-β modulation by LMW is limited, TGF-β signaling is a well-established driver of post-irradiation salivary gland fibrosis [39], suggesting a plausible mechanistic link between LMW’s antioxidant/anti-fibrotic bioactivity and protection of salivary tissues. Macamides in particular have shown various health benefits such as modulating neural function; boosting energy efficiency; promoting bone formation; and providing antioxidant, anti-fatigue, anticancer, hepatoprotective, and anti-memory impairment effects [12,34,40,41,42,43,44]. These findings provide a mechanistic basis for evaluating the therapeutic potential of xerostomia in vivo.

LMWE contains macamides/macaenes, glucosinolates, polyphenols, and polysaccharides, which contribute to antioxidant, anti-inflammatory, and endocrine-modulating effects. Macamides inhibit FAAH and support neuroprotection, while glucosinolates exert anti-oxidative and anti-fibrotic activities. Notably, *L. meyenii* also enhances pituitary gonadotropin secretion in rodents, reflecting its action on secretory glands [30]. Since oxidative stress and TGF-β signaling are central to radiation-induced salivary gland fibrosis, we hypothesized that LMWE, through its antioxidant and anti-fibrotic constituents, may protect salivary glands and improve xerostomia. Using animal, cellular, and organoid models, we examined its effects on salivary secretion, mitochondrial function, and tissue regeneration. To our knowledge, this is the first study to demonstrate the protective and regenerative actions of maca-derived bioactive constituents on salivary glands, highlighting LMWE as a novel dietary-based therapeutic candidate for xerostomia.

## 2. Materials and Methods

### 2.1. Materials

Antibodies against amylase (#3796), Mist1 (#14896), AQP5 (#59558), F4/80 (#70076), and collagen I (#72026) were purchased from Cell Signaling Technology (Danvers, MA, USA). Male C57BL/6J mice (8–10 weeks, 20–25 g) were obtained from the National Laboratory Animal Center (Taipei, Taiwan) under AAALAC accreditation. Pilocarpine (2 mg/kg), Zoletil (50 mg/kg), and xylazine (10 mg/kg) were used for salivary stimulation and anesthesia (Sigma–Aldrich, St. Louis, MO, USA). The Par-C10 rat parotid acinar cell line (originally established by Quissell et al. and kindly provided by Dr. Ching-Yi Wu) was cultured in DMEM/F12 medium (Gibco, Thermo Fisher Scientific, Waltham, MA, USA) supplemented with L-glutamine, N-2 Supplement, dexamethasone, 10% fetal bovine serum, 100 U/mL penicillin, 100 μg/mL streptomycin, human EGF, and human FGF-2. For 3D culture, Matrigel (Corning, #354234, Glendale, AZ, USA) was used. MTT reagent, β-galactosidase staining kit, and ELISA kits for salivary amylase and CRP were obtained from Sigma–Aldrich and Abcam (Cambridge, MA, USA). RNA was reverse-transcribed using the Maxima First Strand cDNA Synthesis Kit (Thermo Fisher Scientific, Waltham, MA, USA), and qPCR was performed with PowerUp SYBR Green Master Mix on a QuantStudio™ 5 Real-Time PCR System (Applied Biosystems, Thermo Fisher Scientific Waltham, MA, USA). Mitochondrial bioenergetics were assessed with a Seahorse XFp Extracellular Flux Analyzer (Agilent Technologies, San Diego, CA, USA), while mitochondrial membrane potential was measured using JC-1 dye on the NucleoCounter^®^ NC-3000™ fluorescence image cytometer (ChemoMetec, Allerød, Denmark). Additional instruments included the Multiskan Sky High microplate spectrophotometer (Thermo Fisher Scientific, Waltham, MA, USA), ECHO Revolve fluorescence microscope (Discover Echo, Inc., San Diego, CA, USA), DRI-CHEM NX500i analyzer (Fujifilm, Tokyo, Japan), and ViewPoint Virtual Slide software (PreciPoint GmbH, Garching, Germany).

### 2.2. Lepidium Meyenii Walpers Extraction (LMWE)

#### 2.2.1. Purchase and Configuration of LMWE

Gelatinized *Lepidium meyenii* Walpers extract (LMWE) in powder form was purchased from 5greens Inc. (https://5greens.co.uk/products/maca-root-capsules-6000mg-0-6-macamides-and-macaenes-500mg-12-1-capsules?srsltid=AfmBOooxpsifQtpq1Fn6rp-xc_01TZ8_byKy6i8XczRtpXLePBrIv8ea, 1 August 2025, an online shop in Manchester, UK). According to the supplier, the product is a gelatinized Maca root extract in a 12:1 concentration, with each capsule containing 500 mg of extract, equivalent to 6000 mg of Maca powder, and containing approximately 0.6% macamides and macaenes. As the extraction procedure and conditions were proprietary to the manufacturer, these details are not available. For experimental use, LMWE powder was dissolved in phosphate-buffered saline (PBS) or Dulbecco’s Modified Eagle Medium/Nutrient Mixture F-12 (DMEM/F12; Thermo Fisher Scientific Inc., Grand Island, NY, USA) to prepare stock solutions at a concentration of 6 mg/mL for subsequent studies.

#### 2.2.2. Analysis and Purification of Macaene Derivatives and Macamides

The LMWE (64 g) was finely ground and extracted three times with dichloromethane by cold maceration, yielding approximately 40 mg of dichloromethane extract. Analytical thin-layer chromatography (TLC) was carried out, and the optimal developing system was determined to be hexane:acetone = 3:1, which was subsequently applied for compound preparation. Both macamides and macaenes, owing to their conjugated double bonds, exhibited distinct absorption spots under short-wavelength UV light (254 nm). Preparative TLC was then performed under the same conditions, and two major UV-absorbing fractions were collected, yielding Maca-1 (1.5 mg) and Maca-2 (0.8 mg). Additional staining of the plates with 1% Ce_2_(SO_4_)_3_ in 10% H_2_SO_4_ followed by heating revealed a minor band (Maca-3, 0.2 mg), although the amount was insufficient for full characterization. Subsequent NMR analyses indicated that Maca-1 corresponded to a macaene derivative and Maca-2 to a macamide class compound, both presumed to be previously unreported. Purified fractions were assessed by analytical HPLC-DAD (C18, water/acetonitrile with 0.1% formic acid, gradient elution), monitoring at 210, 254, and 280 nm. Peak purity was verified by diode-array spectra and expressed as area percentage (acceptance: ≥98%). The purified compounds were dissolved in DMSO to prepare 100 mM stock solutions for subsequent biological assays.

### 2.3. Animal Study

#### 2.3.1. Ethical Approval

Nine-week-old female C57BL/6J mice were purchased from the National Laboratory Animal Center (Taipei, Taiwan), an AAALAC-accredited facility. All animal studies were approved by the Institutional Animal Care Committee of the National Defense Medical University (Certificate No.: IACUC 21-247/approved on 24 September 2021 and Certificate No.: IACUC 23-013/approved on 13 February 2023), Taiwan. Animal care conformed to the “Guide for the Care and Use of Laboratory Animals” published by the U.S. National Institutes of Health (NIH Publication No. 85-23, revised in 1996).

#### 2.3.2. Induction of Salivary Gland Damage in Mice Through γ-Ray Radiation

According to previous reports [45,46], 9-week-old female C57BL/6J mice were used to receive a single 15.0 Gray irradiation of salivary glands (mainly submandibular glands) to establish an animal model of salivary gland hypofunction. Before irradiation, the mice were anesthetized with isoflurane and allowed to lie on their backs and stretch their chins. During the irradiation process, the area other than the salivary glands was shielded using a lead plate. After irradiation, mice were returned to the cage and continued to receive adequate food and water. All procedures were performed at the Taiwan Mouse Clinic and Animal Consortium in Taipei, Taiwan.

#### 2.3.3. Animal Grouping and Treatment

Starting on the day of radiation exposure, LMWE was administered via oral gavage using a feeding syringe once daily. The animal groups were divided as follows: Group 1: Mice not exposed to radiation, sacrificed at 6 and 12 weeks after entering the experiment (*n* = 10); Group 2: Mice exposed to radiation, fed 200 μL of PBS daily, and sacrificed at 6 and 12 weeks after entering the experiment (*n* = 10); Group 3: Mice exposed to radiation, fed 6 mg/kg body weight (BW) of LMWE daily, and sacrificed at 6 and 12 weeks after entering the experiment (*n* = 10); and Group 4: Mice exposed to radiation, fed 12 mg/kg BW of LMWE daily, and sacrificed at 6 and 12 weeks after entering the experiment (*n* = 10). Five experimental mice from each group were sacrificed at 6 weeks (*n* = 5) and 12 weeks (*n* = 5), respectively.

#### 2.3.4. Morphological Analysis

At the end of the experiment (6 and 12 weeks post-radiation exposure), mice were euthanized under deep anesthesia using intraperitoneal Zoletil (50 mg/kg) and xylazine (10 mg/kg), followed by cervical dislocation. This method ensured loss of consciousness and complied with institutional guidelines for humane animal sacrifice. The submandibular glands were harvested and weighed. The glands were then analyzed using hematoxylin and eosin (H&E) staining, Masson’s trichrome staining, immunohistochemistry, and immunofluorescence analysis. Anti-amylase (#3796), anti-muscle, intestine and stomach expression 1 (Mist1; #14896), anti-aquaporin-5 (AQP5; #59558), anti-F4/80 (#70076), and anti-collagen I (#72026) antibodies were purchased from Cell Signaling Inc. (St. Louis, MO, USA). Macrophages and collagen I were visualized by DAB staining, Mist1 was stained with Alexa-594, and AQP5 and amylase were stained with Alexa-488. The acinar cell area (non-striation) to ductal cell area (striation) ratio was calculated using the ViewPoint Virtual Slide Viewing Software (PreciPoint, Garching bei München, Germany) from the 400× magnification images. Collagen fiber accumulation and collagen I content were calculated using the ViewPoint Virtual Slide Viewing Software from the 200× magnification images. The fluorescence was calculated using an ECHO Revolve Hybrid Upright and Inverted Fluorescence Microscope (Echo Laboratories, San Diego, CA, USA).

#### 2.3.5. Analysis of Salivary Lag Time, Saliva Secretion Volume, and Salivary Amylase

Before sacrificing the mice, saliva secretion volume and salivary lag time were analyzed. The mice were anesthetized with isoflurane, and their body weights were measured. Pilocarpine (2 mg/kg), a cholinergic parasympathomimetic agent, was administered via intraperitoneal injection, and the time in seconds required for the onset of salivary secretion after injection was recorded. Simultaneously, a pipette was used to collect saliva from the floor of the mouse’s mouth over a 10 min period. The total amount of saliva collected was defined as the volume of the secreted saliva. Salivary amylase was identified using enzyme-linked immunosorbent assay (Abcam Inc., Waltham, MA, USA).

#### 2.3.6. Biochemical Measurements

Blood samples for biochemical measurements were collected from each animal before and at the end of the experiment. Samples were obtained from the tail vein and collected in tubes containing heparin, and plasma was separated by centrifugation. Plasma samples were stored at −80 °C until further use. Plasma total BUN, creatinine, alanine transaminase (ALT), aspartate aminotransferase (AST), and lactate dehydrogenase (LDH) levels were measured using an automatic Dry Chemistry Analyzer (DRI-CHEM NX500i; Fujifilm, Tokyo, Japan). C-reactive protein (CRP) was measured by enzyme-linked immunosorbent assay (Abcam Inc., Cambridge, MA, USA).

### 2.4. In Vitro Study

#### 2.4.1. Cultivation of Par-C10 Cells and Salivary Organoid Preparation

The original rat parotid acinar cell line (Par-C10) was established by Quissell et al. [47]. Par-C10 cells used in this experiment were kindly provided by Dr. Ching-Yi Wu. According to previous reports, Par-C10 cells was cultured in DMEM/F12 medium including L-glutamine, N-2 Supplement, dexamethasone, fetal bovine serum, human epidermal growth factor (EGF), human fibroblast growth factor basic (FGF-2), and insulin-transferrin-selenium (ITS-G) [48,49]. Par-C10 cells were passaged in culture dishes, and once the cells reached 80% confluence, they were used to prepare and culture 3D organoids. A total of 4 × 10^5^ Par-C10 cells were mixed with 40 μL of Matrigel (cat# 354234; Corning Inc., Corning, NY, USA), then placed in a pre-warmed 35 mm cell culture dish and inverted in a 37 °C incubator. After 60 min, DMEM/F12 medium was added, and the cells were cultured in the 37 °C incubator for 14 days. The differentiation medium used to promote cell differentiation contained L-glutamine, N-2 Supplement, dexamethasone, EGF, FGF-2, and ITS-G. The basal medium (control medium) did not contain any supplements. In this study, experiments were conducted using cells from passages 2 to 12.

#### 2.4.2. 3-(4,5-Dimethylthiazol-2-yl)-2,5-diphenyltetrazolium Bromide (MTT) Assay

According to a previous report, the effect of LMWE on mitochondrial succinate dehydrogenase activity in Par-C10 cells was analyzed using an MTT assay [50]. First, 7 × 10^3^ Par-C10 cells were seeded into a 96-well cell culture plate and cultured for 24 h. Afterward, the cells were treated with LMWE for 24 or 48 h with or without TGF-β treatment. Following the treatment, the cells were incubated with 0.5 mg/mL of MTT solution for 4 h to assess succinate dehydrogenase activity, which reflects mitochondrial function. Optical density (OD) was measured at 570 nm using a Multiskan Sky High microplate spectrophotometer (Thermo Fisher Scientific Inc., Waltham, MA, USA).

#### 2.4.3. Cellular Senescence Assay

A β-galactosidase activity staining kit from Sigma-Aldrich (St. Louis, MO, USA) was utilized to evaluate Par-C10 cell aging [51]. The method involved initially fixing the Par-C10 cells with a solution containing formaldehyde and glutaraldehyde. Following fixation, the cells were incubated in freshly prepared X-gal staining solution for 12 h. After staining, the blue-colored cells and total number of cells were counted to determine the percentage of cells exhibiting β-galactosidase activity.

#### 2.4.4. Transepithelial Electrical Resistance (TEER) Assay for Cellular Barrier Capacity

We used a TEER assay [52] to analyze the effect of LMWE on the barrier capacity of Par-C10 cells. TGF-β and LMWE were incorporated into the cultured Par-C10 cells (3 × 10^4^ cells/wells) in Maestro Edge 96-well plates after ensuring the stability of cell attachment. Maestro Z (AXION BioSystems, Atlanta, GA, USA) was used to continuously monitor changes in electrical resistance for 48 h. The area under the resistance change curve was obtained by integration to represent cellular barrier integrity.

#### 2.4.5. Seahorse XFp Platform for Bioenergetic Analysis

The Seahorse XFp platform (Agilent, San Diego, CA, USA) was used to assess cellular energy metabolism [53]. According to the manufacturer’s instructions, Par-C10 cells at a concentration of 10^4^ cells per well were seeded into the cell plate. The cells were then exposed to TGF-β1 and LMWE, followed by incubation at 37 °C for 24 h. Approximately 1 h prior to analysis, the culture medium was replaced with serum-free medium. Specific reagents including Oligomycin, FCCP, and rotenone/antimycin A, were subsequently added to the probe plate grooves. The Seahorse XFp platform was used to measure mitochondrial oxidative phosphorylation and oxygen consumption in the cellular environment.

#### 2.4.6. Mitochondrial Potential Assay

Mitochondrial transmembrane potential was assessed using JC-1 fluorescent dye (ChemoMetec, Allerød, Denmark) with a NucleoCounter^®^ NC-3000™ fluorescence image cytometer [54]. In viable cells, the negative charge generated by the intact mitochondrial membrane potential facilitates the accumulation of the red fluorescent JC-1 in the mitochondrial matrix. Conversely, in non-viable cells, the collapse of mitochondrial potential causes JC-1 to remain in the cytosol in its monomeric green fluorescent form. Cells with a reduced mitochondrial potential show a decreased red-to-green fluorescence ratio. Par-C10 cells were seeded into 6-well plates, and after a 24 h incubation with TGF-β and LMWE, the collected cell pellets were promptly analyzed using NC-Slides A8 with eight chambers (ChemoMetec, Allerød, Denmark).

#### 2.4.7. Identifying the Markers of Salivary Gland Organoids

The expression of markers in salivary gland organoids was analyzed via H&E and periodic Acid–Schiff (PAS) staining. Additionally, salivary gland organoids were identified using real-time polymerase chain reaction (PCR). Briefly, total RNA was reverse transcribed into complementary DNA (cDNA) using the Maxima First Strand cDNA Synthesis Kit (Thermo Fisher Scientific Inc., Waltham, MA, USA). PCR was then performed using PowerUp™ SYBR™ Green Master Mix (Applied Biosystems; Thermo Fisher Scientific Inc., Waltham, MA, USA). The QuantStudio™ 5 Real-Time PCR System (Thermo Fisher Scientific Inc., Waltham, MA, USA) was used to detect the Ct values of samples, which were applied to a formula to calculate the differences in mRNA expression levels. GAPDH was used as a normalized control. The primers used in this study are listed in Table 1.

### 2.5. Statistical Analyses

Values are expressed as the mean ± standard deviation (SD). One-way analysis of variance (ANOVA) and post hoc tests were used to conduct statistical analyses, followed by Tukey’s test. Statistical significance was set at *p* < 0.05.

## 3. Results

### 3.1. LMWE Primarily Contains (Z)-N-Phenyldodec-2-enamide and (1E,4Z)-1-(2,4-Dihydroxyphenyl)-5-(3,4-dihydroxyphenyl) Penta-1,4-dien-3-one

**Figure 1:**
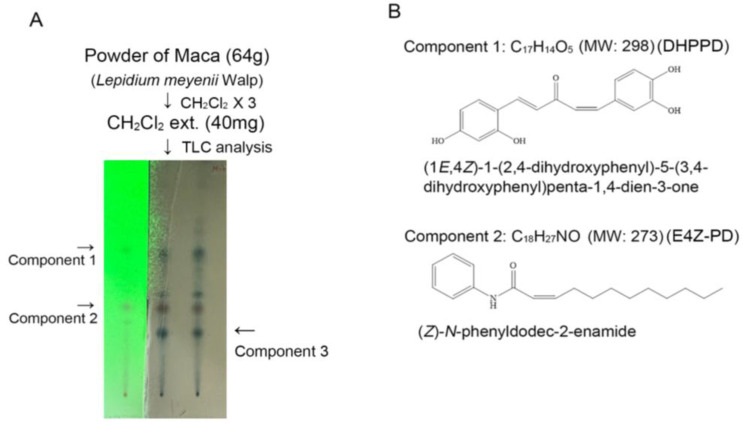
LMWE mainly contains macaene and macamide. (**A**,**B**) Chromatography and NMR analysis identified two major LMWE component 1, macaene [(1*E*,4*Z*)-1-(2,4-dihydroxyphenyl)-5-(3,4-dihydroxyphenyl)penta-1,4-dien-3-one] and component 2, macamide [(*Z*)-*N*-phenyldodec-2-enamide].

We analyzed the components of LMWE using Prep TLC and identified their structures with NMR. As shown in Figure 1A, LMWE displayed two distinct spots, spot 1 (component 1/1.5 mg) and spot 2 (component 2/0.8 mg), under 254 nm UV light after Prep TLC development. An additional compound, spot 3 (component 3/0.2 mg), was detected after spraying the TLC plate with 1% Ce_2_(SO_4_)_3_ in 10% H_2_SO_4_ aqueous solution followed by heating, which produced a charred coloration. Following NMR analysis and comparison with the literature, we hypothesized that component 1 was a macaene derivative, (1*E*,4*Z*)-1-(2,4-dihydroxyphenyl)-5-(3,4-dihydroxyphenyl) penta-1,4-dien-3-one (DHPPD), and component 2 was compounds of macamide class, (*Z*)-*N*-phenyldodec-2-enamide (E4Z-PD). Maca-3 was obtained in too small an amount for further structural elucidation. These two structures have not been previously reported in LMWE (Figure 1B). Based on their structural formulas, the molecular weights of the two compounds were calculated as 298 and 273, respectively.

### 3.2. Administration of LMWE Did Not Affect the Biochemical Characteristics in C57BL/6 Mice

Biochemical analyses were performed to determine the toxicity of LMWE in C57BL/6 mice. Additionally, plasma LDH and CRP levels were measured to assess the protective effects of LMWE against irradiated salivary gland injury. As shown in Table 2, body weight changes, kidney function indicators (BUN and creatinine), liver function indicators (ALT and AST), and CRP levels did not differ between the groups during the experimental period. LDH is an enzyme involved in glucose metabolism that is widely present in various organs. When cells are damaged, plasma LDH levels increase. As shown in Table 1, plasma LDH levels increased 6 weeks after radiation exposure (198.4 ± 62.7 IU/L), although this increase was not significantly different from pre-exposure levels (90.8 ± 27.7 IU/L). The LMWE-fed groups appeared to show a trend toward reducing LDH levels (123.5 ± 56.1 IU/L in the radiation injury + LMWE 6 mg/kg BW group and 135.7 ± 43.1 IU/L in the radiation injury + LMWE 12 mg/kg BW group), although there was still no significant difference compared with the radiation injury group. After 12 weeks of radiation exposure, the plasma LDH levels in all groups returned to baseline values. These data indicate that radiation exposure to the salivary glands and LMWE feeding did not affect liver and kidney function or the inflammatory marker CRP. Radiation exposure to the salivary glands only caused non-significant fluctuating changes in LDH, a marker of tissue damage.

### 3.3. LMWE Reversed Reduction in Saliva Secretion and Prolonged Secretion Lag Time in Radiation-Exposed C57BL/6 Mice

**Figure 2:**
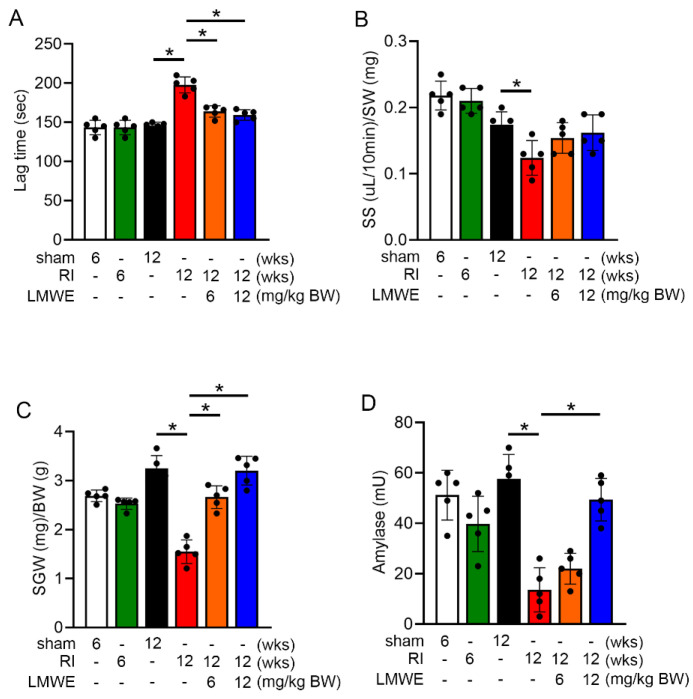
LMWE can mitigate the radiation-induced delay in saliva secretion and decrease in amylase activity in C57BL/6 mice. (**A**) At the end of the experiment, secretion lag time after pilocarpine injection was measured. (**B**) Saliva was continuously collected for 10 min from the start of secretion (μL/10 min). The total volume of saliva secreted (SS) was divided by the salivary gland weight (SGW) to compare differences between groups. (**C**) The salivary gland weight (SGW) was divided by body weight (BW) to compare group differences. (**D**) Amylase activity in the saliva of each experimental group was analyzed by ELISA. All results in the graphs are expressed as the mean ± SD (*n* = 5). * Statistical significance was set at *p* < 0.05.

At the end of the experiment, the time until saliva secretion began after pilocarpine injection (secretion lag time) was measured along with the volume of saliva secreted over the subsequent 10 min. After sacrificing the mice, the parotid glands were removed and weighed. As shown in Figure 2A, radiation exposure for 12 weeks significantly increased lag time. Feeding LMWE effectively reduced this prolonged lag time (sham control [12 weeks] group: 145.8 ± 3.8 s, radiation injury [12 weeks] group: 197.8 ± 9.2 s, radiation injury + LMWE 6 mg/kg BW group: 163.8 ± 6.5 s, and radiation injury + LMWE 12 mg/kg BW group: 159.4 ± 6.1 s). Figure 2B shows a significant reduction in total saliva secretion 12 weeks after radiation exposure. Although LMWE treatment tended to increase total saliva secretion, the increase was not statistically significant compared with that in the radiation injury group. However, treatment with LMWE (6 and 12 mg/kg BW) significantly increased the weight of the salivary glands compared to the radiation injury group (Figure 2C). Additionally, treatment with LMWE (12 mg/kg BW) significantly increased amylase activity in the salivary glands of radiation-exposed C57BL/6 mice (Figure 2D). These data indicate that functional loss of the salivary glands appears at 12 weeks after radiation exposure in mice, and LMWE can mitigate the radiation-induced delay in saliva secretion and decrease in amylase activity, although the trend towards increased saliva secretion did not reach statistical significance.

### 3.4. LMWE Protected Acinar Cells in Radiation-Exposed C57BL/6 Mice

**Figure 3:**
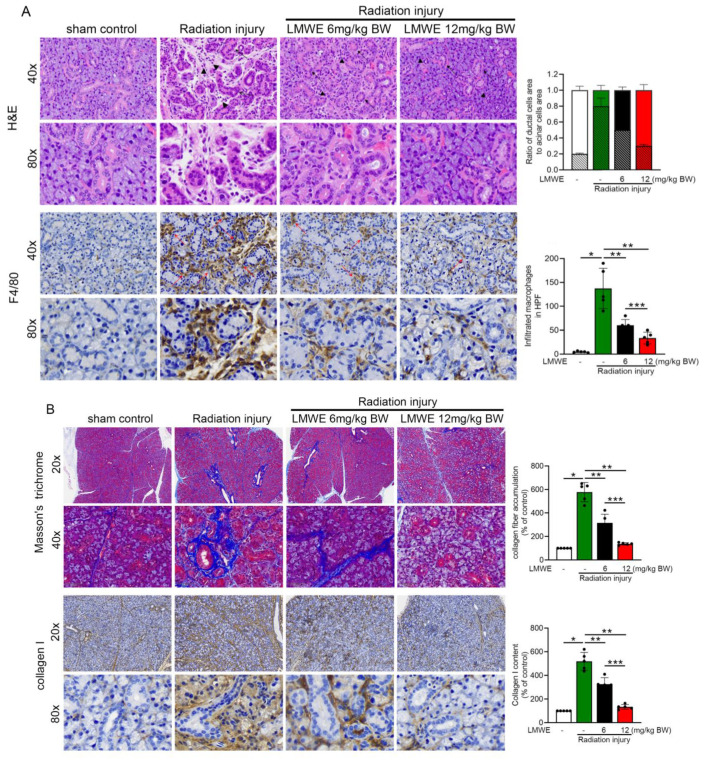

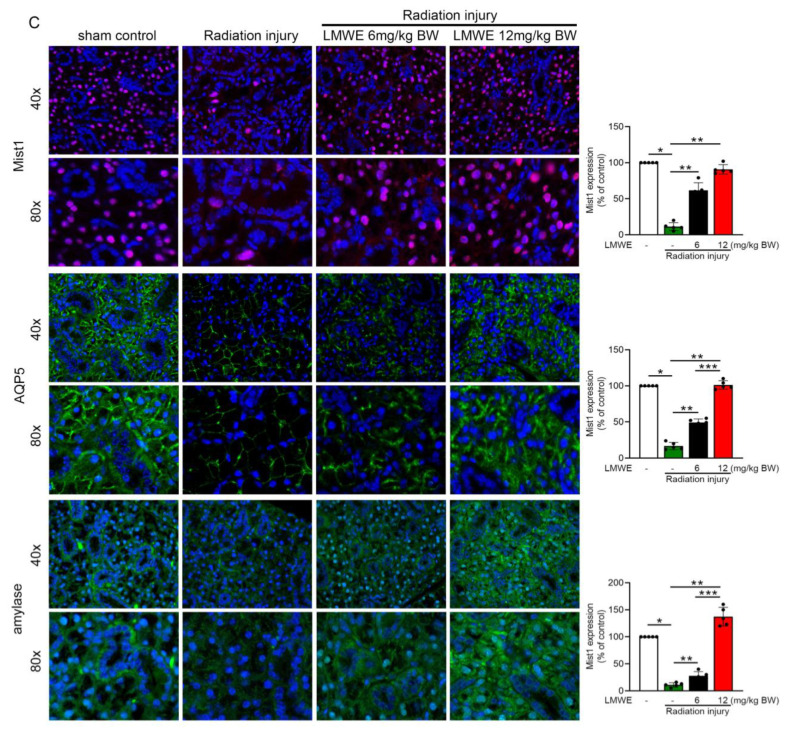
LMWE protected acinar cells in radiation-exposed C57BL/6 mice. (**A**) Representative images of H&E and F4/80 staining showing acinar cell atrophy, ductal dilation, and macrophage infiltration. Quantification of acinar/ductal ratio and macrophage counts is shown in the bar graphs. (**B**) Masson’s trichrome and collagen I staining showing radiation-induced fibrosis and its attenuation by LMWE treatment. Quantification of collagen deposition is shown in the bar graphs. (**C**) Immunofluorescence images of Mist1, AQP5, and amylase expression in salivary glands. Quantification of fluorescence intensity is presented in the bar graphs. Data are expressed as mean ± SD (*n* = 5). * *p* < 0.05 vs. control; ** *p* < 0.05 vs. radiation injury; *** *p* < 0.05 vs. LMWE 6 mg/kg.

At the end of the experiment (12 weeks), the salivary glands were removed for morphological analysis to examine the effect of LMWE. In Figure 3A, H&E staining (upper column) shows that compared with the sham control group, the radiation injury group at 12 weeks post-exposure exhibited significant submandibular gland tissue damage, including acinar cell (black arrowheads) atrophy, a marked reduction in the number of acinar cell nuclei, widened interacinar spaces, proliferation of small ducts, and intercalated duct (black arrows) dilation. Software was used to calculate the areas covered by acinar and ductal cells, and the results are displayed in the bar graph on the right. The results showed that radiation injury reduced the area of acinar cells while increasing the area of ductal cells. LMWE treatment reversed this change in the acinar-to-ductal cell area ratio, with the effect being most pronounced in the 12 mg/kg BW LMWE treatment group. F4/80 staining revealed substantial macrophage infiltration (red arrows). In the LMWE treatment groups (6 and 12 mg/kg BW), improvements in radiation-induced submandibular gland tissue damage were observed, with the 12 mg/kg BW LMWE treatment restoring the damaged salivary gland morphology to a level comparable to that of the sham control group. Fibrosis can occur after salivary gland injury; therefore, we used immunohistochemistry to observe collagen I content and Masson’s trichrome staining to analyze collagen fiber accumulation in the tissues. As shown in Figure 3B, radiation injury increased collagen fiber accumulation (blue signaling in Masson’s trichrome staining) and collagen I expression (brown signaling in collagen I staining) in the salivary gland tissue, whereas LMWE treatment at both 6 mg/kg BW and 12 mg/kg BW released the expression of collagen I and accumulation of collagen fiber. Mist1 is a protein that is specifically expressed in secretory cells and is located in the nucleus. AQP5 is a water channel protein that is highly expressed in the salivary glands. Amylase is a salivary hydrolase that converts starch into sugar. When the acinar cells in the salivary glands are damaged, Mist1, AQP5, and amylase levels decrease. As shown in the immunofluorescence images in Figure 3C, radiation exposure inhibited the expression of Mist1, AQP5, and amylase. LMWE treatment reversed the radiation-induced decrease in Mist1, AQP5, and amylase expression. Overall, LMWE alleviated the radiation-induced salivary gland damage.

### 3.5. LMWE Survives Mitochondrial Membrane Potential and Function to Prevent Par-C10 Cell Function in TGF-β-Stimulated Situation

**Figure 4:**
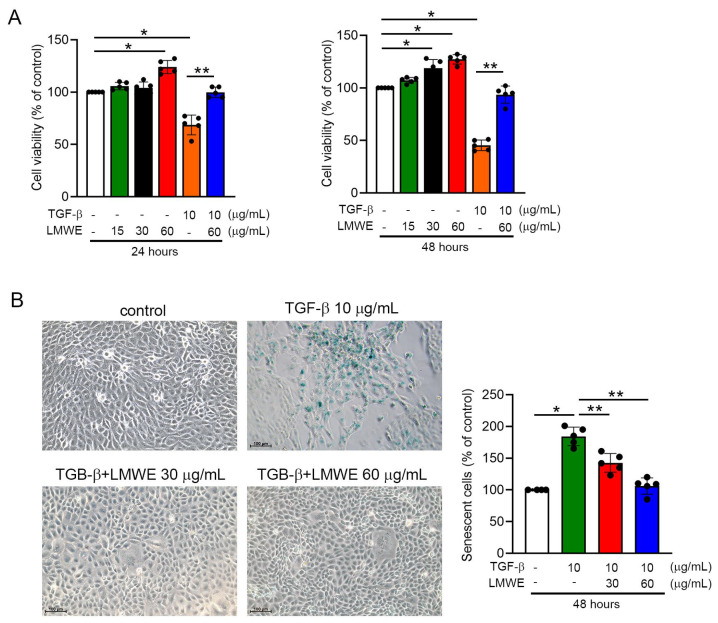

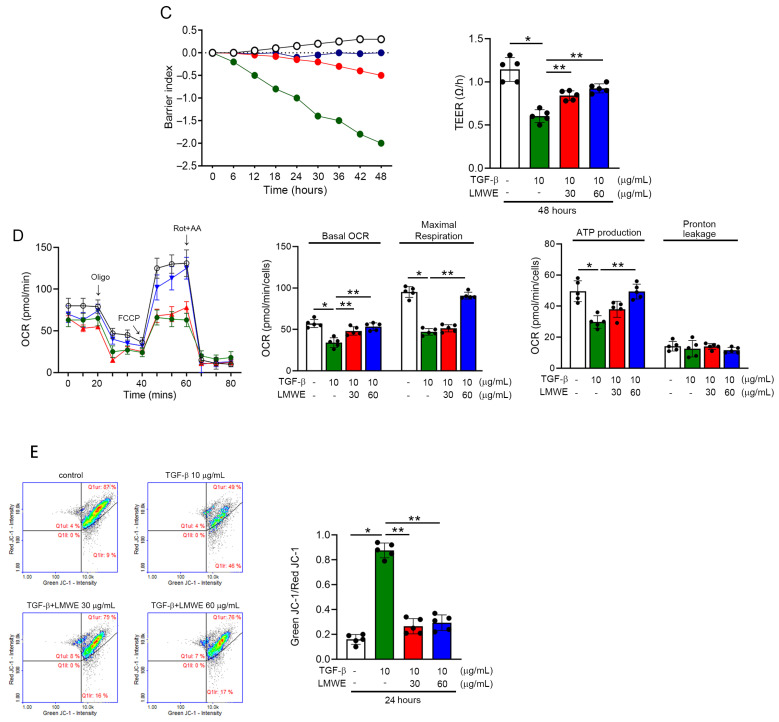
LMWE survives mitochondrial membrane potential and function to prevent senescence in TGF-β1-cultured Par-C10 cells. (**A**) MTT assay showing the effects of LMWE on mitochondrial activity in Par-C10 cells with or without TGF-β1 stimulation. (**B**) Representative images of β-galactosidase staining showing reduced cellular senescence with LMWE treatment, with quantification in the bar graph. (**C**) Transepithelial electrical resistance (TEER) assay demonstrating that LMWE preserved barrier function in TGF-β1–stimulated cells. (**D**) Seahorse analysis of oxygen consumption rate (OCR), showing LMWE rescued basal respiration, maximal respiration, and ATP production compared with TGF-β1 alone. In the upper panel, arrows indicate the specific time points when oligomycin (Oligo), carbonyl cyanide-p-trifluoromethoxyphenyl hydrazone (FCCP), or a combination of rotenone and antimycin A (Rot + AA) were added. (**E**) JC-1 staining showing LMWE preserved mitochondrial membrane potential in a dose-dependent manner. White: control; Green: TGF-β; Red: TGF-β+LMWE 30 μg/mL; Blue: TGF-β+LMWE 60 μg/mL. Data are expressed as mean ± SD (*n* = 5). * *p* < 0.05 vs. control; ** *p* < 0.05 vs. TGF-β1 alone.

The MTT assay results show the effects of TGF-β and LMWE treatment on the viability of Pro-C10 cells at 24 and 48 h. At both time points, treatment with LMWE alone (30 and 60 μg/mL) significantly increased cell viability compared to the control group, suggesting a proliferative or protective effect of LMWE. In contrast, treatment with TGF-β (10 μg/mL) alone significantly reduced cell viability, indicating cytotoxic or growth inhibitory effects. However, co-treatment with LMWE (60 μg/mL) and TGF-β partially restored cell viability compared to TGF-β treatment alone, suggesting that LMWE can mitigate the negative effects of TGF-β (Figure 4A). Additionally, as shown in Figure 4B, Par-C10 cells were treated with 10 μg/mL TGF-β, and senescence analysis was performed 24 h after treatment. TGF-β1 induces the senescence of Par-C10 cells. However, compared with that in the TGF-β1 treatment group, the percentage of senescence-associated β-galactosidase-positive Par-C10 cells was significantly decreased in the LMWE (both 30 and 60 μg/mL) treatment. Barrier capabilities are the basic function of Par-C10 cells in maintaining epithelial polarity. Therefore, TEER can be used as a marker of Par-C10 cell viability. We cultured Par-C10 cells in Maestro Edge plates, exposed them to TGF-β1 with or without LMWE treatment, and measured barrier integrity over time by TEER. In Figure 4C (upper), 10 μg/mL TGF-β1 induced a downtrend in TEER. Additionally, both 30 μg/mL and 60 μg/mL LMWE broke the transition in the TGF-β1 group. Quantification of TEER by integral calculation of the area under the curve and the time of transition (50% drop in barrier index) in Figure 4C (lower) demonstrated that TGF-β1 decreased the barrier capacity. LMWE treatment prevented the negative effects of TGF-β1 on Par-C10 cells. The collective results show that LMWE enhanced the barrier capabilities of TGF-β1-treated Par-C10 cells. Mitochondrial function influences the cellular biological activity by reducing mitochondrial respiration and energy generation during senescence. Therefore, we performed the Seahorse XFp platform to assess the effects of LMWE on mitochondrial function within TGF-β1-treated Pro-C10 cells. Figure 4D illustrates the changes in the oxygen consumption rate (OCR) within Pro-C10 cells over time. Throughout the analysis, oligomycin (oligo), FCCP, and rotenone + antimycin A (Rot + AA) were introduced sequentially. Seahorse XFp analysis software (WAVE ver. 2.6.3) was used to compute metrics, such as basal OCR, maximal respiration, ATP production, and proton leakage. TGF-β1 treatment of 10 μg/mL consistently decreased basal OCR, maximal respiration, and ATP production in Pro-C10 cell mitochondria. Furthermore, treating cells with 60 μg/mL LMWE would reverse the impacts of TGF-β1 on basal OCR, maximal respiration, and ATP production. As a change in the mitochondrial membrane potential is an early phenomenon of cell aging and abnormal respiration, we analyzed this change to observe the effect of LMWE on Pro-C10 cells. In Figure 4E, TGF-β1 increased the green JC-1/red JC-1 ratio, indicating that the mitochondrial membrane potential decreased in Pro-C10 cells at 10 μg/mL TGF-β1 treatment. LMWE treatment at 30 and 60 μg/mL may be effective against decreasing mitochondrial membrane potential in TGF-β1-treated Pro-C10 cells. These results indicate that LMWE promotes normal mitochondrial membrane potential and prevents senescence in TGF-β1-cultured Pro-C10 cells.

### 3.6. LMWE Accelerates the Differentiation of Par-C10 Cell-Derived Organoids

**Figure 5:**
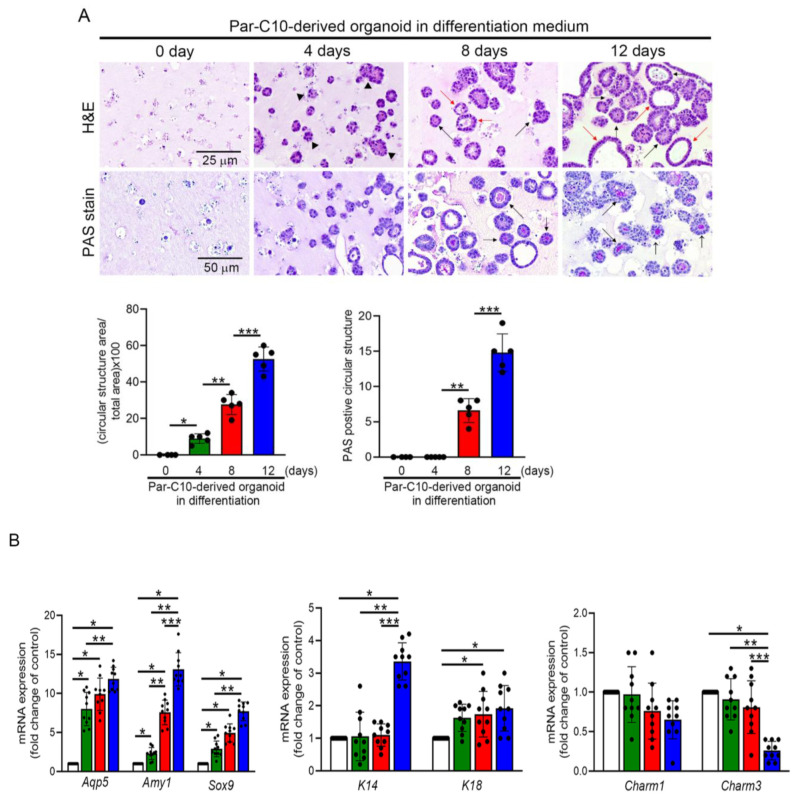

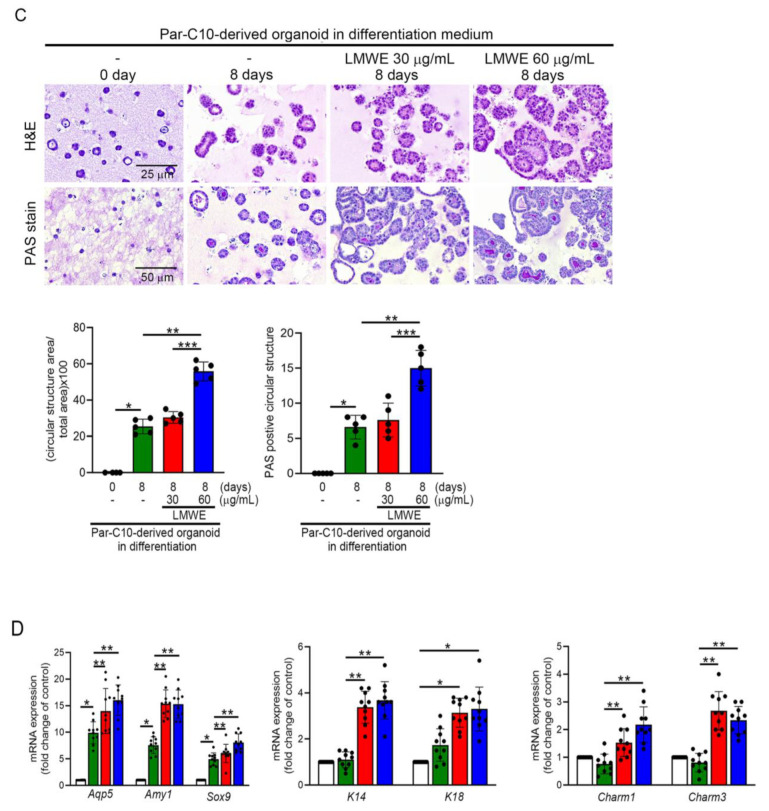
LMWE accelerates the differentiation of Par-C10 cell-derived organoids. Par-C10 cells were embedded in Matrigel and cultured to form organoids with or without LMWE treatment. (**A**,**C**) H&E and PAS staining showing acinar-like (black arrows) and ductal-like (red arrows) structures, as well as PAS-positive signals. Quantification of acinar- and ductal-like structures and PAS-positive areas are shown in bar graphs (*n* = 5). (**B**,**D**) mRNA expression of acinar and ductal markers (Aqp5, Amy1, Sox9, K14, K18, Charm1, and Charm3) in organoids cultured for the indicated days with or without LMWE (*n* = 10). Data are presented as mean ± SD. * *p* < 0.05 vs. control; ** *p* < 0.05 vs. 4- or 8-day groups; *** *p* < 0.05 vs. LMWE 30 μg/mL group.

Under 2D culture conditions, LMWE effectively prevents senescence and maintains mitochondrial activity in Par-C10 cells under TGF-β stimulation. To explore this further, Par-C10 cells were encapsulated in Matrigel and cultured in a suspension-based 3D system to induce salivary gland organoids, and the effect of LMWE on Par-C10 cell differentiation was analyzed. In the upper column of Figure 5A, the H&E staining results show that by day 4 of culture, the internal cells began to aggregate into clusters (black arrowheads). By day 8, the cell clusters had grown larger and showed an organized arrangement, forming acinar- (black arrows) and ductal-like structures (red arrows). By day 12, the aggregation of internal cells further increased, and the formation of acinar- and ductal-like structures became more pronounced and stable. Additionally, PAS staining was used to analyze the ability of the organoids to secrete functional glycoproteins and mucins. In the PAS staining results (lower column of Figure 5A), while slight cell aggregation was observed on day 4, almost no PAS staining signals were detected. By day 8, purple-red signals (black arrows) had appeared in the luminal areas of the acinar- and ductal-like structures. These signals intensified as the culture progressed to day 12. Real-time PCR was used to evaluate the gene expression profiles of 3D-cultured salivary gland organoids. The analyzed genes included acinar cell markers (*Aqp5* and α-amylase (AMY1)), progenitor cell markers (K14 and *Sox9*), a ductal cell marker (K18), and muscarinic acetylcholine receptors (Charm1 and Charm3). As shown in Figure 5B, 3D suspension culture significantly increased the mRNA expression of *Aqp5*, *Amy1*, and *Sox9* as early as day 4 of culture. However, the expression of K14 and K18 mRNA required at least 8 days of 3D suspension culture to increase. In contrast, Charm1 and Charm3 mRNA expression decreased during 3D suspension culture. These observations highlight the progressive differentiation and functional maturation of Par-C10 cells into salivary gland organoids during the 12-day 3D culture period. This suggests that, while 3D culture promotes differentiation into acinar and ductal cells, it suppresses the expression of muscarinic acetylcholine receptors. As shown in Figure 5C, treatment with LMWE at 30 μg/mL for 8 days was insufficient to promote the formation of tubular-like structures or to increase glycoprotein and mucin production in cell clusters compared to the untreated group. However, increasing the LMWE concentration to 60 μg/mL clearly enhanced the formation of tubular-like structures and the secretion of glycoproteins and mucin. The real-time PCR results showed that LMWE treatment at 30 μg/mL and 60 μg/mL increased the mRNA expression of *Aqp5*, *Amy1*, *Sox9*, K14, Charm1, and Charm3 compared to the non-LMWE treatment group. Although K18 mRNA expression showed an increasing trend, it was not statistically significant. Collectively, these findings suggest that LMWE treatment, particularly at a higher concentration of 60 μg/mL, can effectively enhance tubular-like structure formation, functional glycoprotein/mucin secretion, and the expression of key differentiation markers, further supporting its role in promoting salivary gland cell differentiation and maturation.

### 3.7. DHPPD and E4Z-PD Contained in LMWE Exhibit Different Effects on the Differentiation of Praa-C10 Cell-Derived Organoids

**Figure 6:**
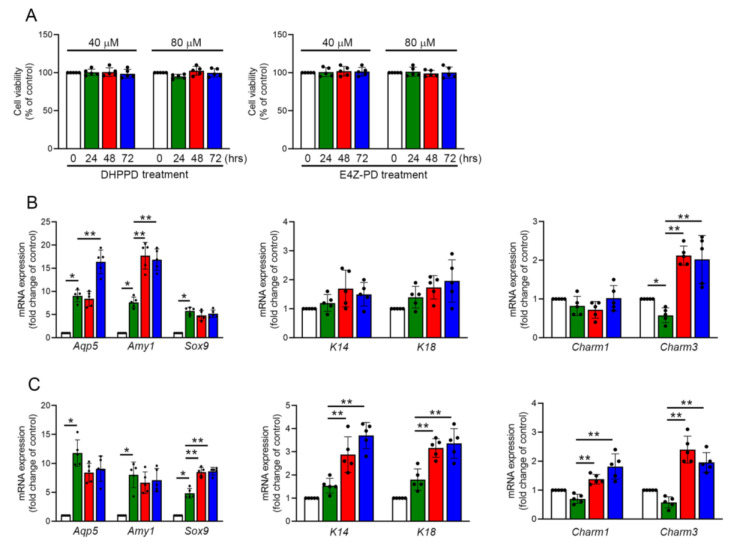
DHPPD and E4Z-PD exhibit different effects on Pra-C10 cell-derived organoids (**A**) MTT assay showing the effects of DHPPD or E4Z-PD on Par-C10 cell viability. (**B**,**C**) Real-time PCR analysis of acinar and ductal markers (Aqp5, Amy1, Sox9, K14, K18, Charm1, Charm3) in organoids treated with DHPPD or E4Z-PD for 8 days. Data are presented as mean ± SD. * *p* < 0.05 vs. control; ** *p* < 0.05 vs. 8-day group (*n* = 5).

Previous results demonstrated that LMWE promotes the differentiation of Par-C10 cell-derived organoids. We further analyzed the differences in how the two major components of LMWE, DHPPD and E4Z-PD, promote organoid differentiation. We first used an MTT assay to evaluate its toxicity in 2D-cultured Par-C10 cells. In Figure 6A, treatment with 40 μM and 80 μM of DHPPD or E4Z-PD for 24–72 h did not have any negative effects on the survival or viability of Par-C10 cells. As shown in Figure 6B, DHPPD treatment promoted the mRNA expression of *Aqp5*, *Amy1*, and Charm3 in Par-C10 cell-derived organoids but had no significant effect on the expression of *Sox9*, K14, K18, and Charm1 mRNA. In contrast (Figure 6C), E4Z-PD treatment increased the mRNA expression of *Sox9*, K14, K18, Charm1, and Charm3 but had no effect on the expression of *Aqp5* or *Amy1* mRNA. Based on these results, we concluded that DHPPD treatment promoted the differentiation of acinar cells, while E4Z-PD treatment enhanced the progenitor cell characteristics of Par-C10 cells and directed them toward ductal cell differentiation. Additionally, E4Z-PD stimulated the expression of cholinergic receptors in these cells in 3D organoids. A novel and noteworthy finding of this study was the identification of two active components within LMWE-DHPPD and E4Z-PD, which differentially promote acinar and ductal lineage differentiation, respectively. This lineage-specific activity provides unique insights into how natural compounds can guide epithelial cell fate and suggests the potential of using LMWE-derived molecules to selectively regenerate salivary gland compartments. This approach may prove valuable in tissue engineering, where the ability to direct the formation of acinar or ductal structures can greatly enhance the design of functional salivary organoids.

## 4. Discussion

In this study, LMWE significantly reduced the radiation-induced delay in salivary secretion and restored both gland weight and amylase activity, indicating preservation of glandular function. However, the overall increase in saliva volume did not reach statistical significance. This discrepancy may reflect irreversible structural damage at the chronic phase of irradiation, limited sample size, or inherent biological variability. From a translational perspective, the absence of a robust improvement in total salivary output may constrain LMWE’s direct applicability for symptomatic relief of xerostomia, since patients often value perceptible increases in oral moisture. Nevertheless, salivary quality, including enzymatic activity, antimicrobial capacity, and buffering properties, is equally critical for oral health. The observed recovery of amylase activity and functional markers such as AQP5 suggests that LMWE not only improves secretion kinetics but also enhances the protective and digestive functions of saliva. Thus, while LMWE alone may not fully normalize salivary volume, its multifaceted benefits support its potential as a protective or adjunctive therapeutic strategy for radiation-induced xerostomia. Future investigations in larger animal cohorts and clinical studies should clarify whether combining LMWE with other sialagogues could achieve both quantitative and qualitative restoration of salivary function.

Given the limitations of current pharmacological treatments for xerostomia, the development of new drugs or natural compounds with fewer side effects to restore salivary gland function has become a key focus of ongoing research. This study demonstrated the multifaceted protective effects of LMWE against radiation-induced salivary gland injury. Unlike amifostine, which primarily acts through free radical scavenging and thiol-based cytoprotection, LMWE offers broader cellular benefits, including preservation of mitochondrial membrane potential, enhancement of epithelial barrier integrity, and reduction in radiation-induced cellular senescence. These additional protective mechanisms suggest that LMWE may serve as a complementary or alternative to amifostine, particularly in clinical contexts where the systemic side effects of current therapies remain challenging. Additionally, a novel and noteworthy finding of this study was the identification of two active components within LMWE-DHPPD and E4Z-PD, which differentially promote acinar and ductal lineage differentiation, respectively. This lineage-specific activity provides unique insights into how natural compounds can guide epithelial cell fate and suggests the potential of using LMWE-derived molecules to selectively regenerate salivary gland compartments. This approach may prove valuable in tissue engineering, where the ability to direct the formation of acinar or ductal structures can greatly enhance the design of functional salivary organoids. Additionally, from a translational standpoint, LMWE may serve two complementary roles in clinical practice. As a standalone therapy, LMWE could provide a low-toxicity, plant-derived alternative to amifostine, particularly for patients who cannot tolerate the systemic adverse effects of current pharmacological agents. However, given that LMWE did not significantly increase overall saliva volume, its monotherapy use may primarily benefit patients whose xerostomia is characterized by qualitative rather than quantitative dysfunction. As an adjuvant therapy, LMWE may be more impactful when combined with sialogogues such as pilocarpine or with established radioprotectants like amifostine. In this setting, LMWE’s ability to preserve glandular architecture, enhance enzymatic activity, and reduce fibrosis could synergize with agents that stimulate fluid secretion, thereby achieving both functional restoration and symptom relief. Overall, these findings support a dual conceptualization of LMWE: first, as a safer botanical candidate for patients requiring long-term supportive care, and second, as an adjunctive therapy capable of amplifying the efficacy of conventional treatments. In animal studies, we selected LMWE doses of 6 and 12 mg/kg to maintain clinical relevance, as 12 mg/kg in mice corresponds to a human-equivalent dose of ~1.0 mg/kg, well below the gram-level daily intake tolerated in clinical trials [55,56]. The 12 mg/kg dose consistently yielded superior renal functional and histological outcomes, likely reflecting stronger engagement of antioxidant and anti-inflammatory pathways [57]. Although preclinical and clinical studies suggest a wide safety margin for maca extracts [58,59,60], rare reports of hepatotoxicity with non-standardized products highlight the need for further GLP toxicology to establish an LMWE-specific NOAEL before clinical translation [61]. Future clinical studies should systematically evaluate LMWE’s comparative efficacy, safety, and combinatorial potential with existing therapies to clarify its role in standard xerostomia management protocols.

In the present study, gelatinized maca root was selected rather than preparing an ethanolic extract from raw plant material. Gelatinisation, a traditional processing method involving controlled heating, disrupts starch granules and enhances the digestibility and bioavailability of maca constituents, while reducing gastrointestinal adverse effects associated with raw maca powder [62,63]. Furthermore, the use of standardized commercial gelatinized maca provides consistent quality for experimental reproducibility, whereas laboratory-prepared ethanolic extracts may vary with extraction conditions. Finally, since gelatinized maca capsules are widely marketed as dietary supplements, our approach enhances the translational relevance of the findings, directly reflecting the form in which patients are most likely to consume maca. In addition to the observed bioactivities, our chemical analysis isolated two conjugated compounds from LMWE, designated Maca-1 and Maca-2. NMR characterization identified Maca-1 as DHPPD (a macaene derivative) and Maca-2 as E4Z-PD (a macamide). To verify their novelty, we performed a comprehensive search in the Reaxys database (https://www.reaxys.com), which yielded 146 previously reported constituents of *L. meyenii*. Neither Maca-1 nor Maca-2 was found among these compounds, supporting the conclusion that they represent newly reported constituents of maca. This finding expands the current phytochemical profile of *L. meyenii* and provides new insights into potential bioactive components that may contribute to its radioprotective and regenerative effects. Further studies are required to validate their biological relevance and to elucidate their mechanisms of action.

Currently, the most commonly used immortalized salivary gland cell lines for generating artificial salivary gland organoid models include HSY, HSG, SMIE, RSMT-A5, SMG-C6, SMG-C10, Par-C10, and Par-C5. Among them, HSY and HSG are derived from human tumor tissue [64]. These two cell lines exhibit well-organized cell polarity, can simulate the structural characteristics of the salivary glands, and possess the ability to secrete saliva and amylase, making them suitable for studies on salivary secretion mechanisms [65,66]. However, because HSY originates from tumor cells, it cannot fully represent the physiological properties of normal salivary gland cells [67,68]. Additionally, HSG lacks the expression of the water channel proteins AQP1 and AQP5, making it an unsuitable model for water transport studies [69]. SMG-C6 and SMG-C10 are derived from normal rat submandibular glands and immortalized using the SV40 virus [47]. Both cell lines demonstrate acinar cell polarity and the ability to form tight junctions and desmosomes [70,71]. Specifically, SMG-C6 responds well to β-adrenergic receptor stimulation and is suitable for studying acinar and ductal structures, whereas SMG-C10 expresses epithelial sodium channels and is ideal for Na^+^ transport-related studies. However, both cell lines exhibit limited differentiation potential in 3D culture environments [72,73]. Par-C5 and Par-C10 originate from normal rat parotid glands [74]. These cells form secretory granules, tight and intermediate junctions, desmosomes, and microvilli [47]. Nevertheless, under 2D conditions or 3D culture with Matrigel encapsulation, the cells exhibit low amylase expression, indicating the need for further optimization to enhance their secretory functions. Par-C10 cells are widely used because of their capacity to form polarized epithelial monolayers. Previous studies have reported that when encapsulated in Matrigel and cultured in 3D, Par-C10 cells can generate organoids that express epithelial markers, such as ZO-1 (tight junction protein), acinar cell marker AQP3 (water channel protein), and M3 muscarinic acetylcholine receptor [74]. These features suggest that Par-C10-derived 3D organoids recapitulate the structural and molecular characteristics of salivary gland acini. Furthermore, a study demonstrated that Par-C10-derived organoids exhibit polarized acinar epithelial architecture, and upon stimulation with the parasympathomimetic agent carbachol, they exhibit a robust intracellular calcium release response [48]. Collectively, these findings suggest that Par-C10 cells when cultured in 3D can reliably form acinar-like organoids, making them a valuable in vitro model for salivary gland research. The Par-C10-derived 3D organoid system used in this study closely mimicked the structural and functional features of native salivary gland tissue, reinforcing the relevance of our findings. The capacity of LMWE to enhance epithelial marker expression and support calcium signaling upon stimulation further highlights its regenerative potential.

In most mammals, salivary gland development is divided into three major stages. The first stage is the initial development stage, where on embryonic day (E) 11.5, epithelial thickening over the neural crest-derived mesenchyme is observed. By E12, an epithelial placode forms and expands into the bud stage by E12.5, initiating ductal and acinar cell differentiation. The second stage is the branching morphogenesis stage, starting at E13, when epithelial–mesenchymal interactions lead to the formation of a branched architecture [75]. By E15, structures composed of KRT19^+^ ductal cells and AQP5^+^ acinar cells emerge, followed by the appearance of α-SMA^+^ myoepithelial cells and expression of secretory-related proteins such as MIST1 and parotid secretory protein (PSP) at E16 [76]. The third stage, maturation, extends from birth to approximately 4 weeks postnatally, during which the gland reaches full structural and functional development, forming serous or mucous acinar cells, ductal cells, and myoepithelial cells capable of producing sufficient saliva, including amylase. Structurally, the mature gland consists of acinar cells (marked by AQP5, AQP3, α-amylase, and MIST1) responsible for saliva secretion, ductal cells (marked by KRT5, KRT14, KRT18, and KRT19) that regulate ion exchange, and myoepithelial cells (marked by α-SMA and p63) that contract to facilitate secretion [77,78]. Throughout development, several progenitor and stem cell populations, including K14, K5, Sox2, Sox9, Sox10, and p63 [79], are essential for differentiation, morphogenesis, regeneration, and tissue maintenance. K14 and K5 are broadly expressed during the early stages and later become restricted to the basal ductal and myoepithelial cells. These markers identify cells with stem-like properties that can differentiate into various epithelial subtypes [80]. Sox2 is widely expressed in early epithelial cells and later localizes mainly to acinar cells, where it promotes differentiation and maintains cellular pluripotency, even in mature glands [81]. Both Sox9 and Sox10 are involved in the differentiation of the acinar and ductal lineages. Sox9 begins expression around E12–E13 and plays a central role during branching morphogenesis, whereas Sox10 marks progenitor cells with high plasticity, contributing to acinar regeneration following injury. Previous studies suggested that Sox9 and Sox10 synergistically promote functional secretory cell differentiation [82]. Additionally, Trp63 (p63) is expressed in basal progenitor cells as early as E10.5 and is involved in the development of both ductal and acinar lineages. During injury repair, p63^+^ cells retain the ability to differentiate into secretory acinar cells, highlighting their regenerative potential. Together, these markers not only define cellular identity during development, but also provide crucial insights for developing salivary gland regeneration strategies.

A growing clinical literature documents herbal products used for xerostomia—particularly in head-and-neck cancer survivors—including plant-derived or natural products have also shown signals of benefit; for example, a recent review highlights thyme honey as a feasible option during and after radiotherapy, and an RCT in non-cancer xerostomia found malic acid and betaine mouthrinses comparably improved dry-mouth symptoms and oral-health-related quality of life [25,83]. Mechanistically, our LMWE contains glucosinolates/isothiocyanates, polyphenols, polysaccharides, and fatty-acid amide derivatives (macaenes/macamides); these classes map onto known antioxidant, anti-inflammatory, and anti-fibrotic pathways that are relevant to radiation-induced salivary-gland injury. In cruciferous plants broadly (e.g., broccoli, radish), glucosinolate-derived isothiocyanates activate Nrf2 and suppress NF-κB signaling and fibrosis, offering a clear pharmacological parallel to LMWE’s constituents; this overlap provides a rationale to compare maca with other Brassicaceae-derived agents in xerostomia [84]. In addition, macamides—largely unique to maca—inhibit FAAH, a target linked to neuro-immune modulation and redox homeostasis; natural-product FAAH modulators are reported across species, but macamide-type N-benzyl long-chain amides are characteristic of Lepidium meyenii, underscoring a compositional distinction from most herbal formulas evaluated for xerostomia [27,85]. Taken together, aligning LMWE’s bioactive chemistry with pharmacology observed in other plant extracts helps explain our multi-model findings (secretion, mitochondrial support, anti-fibrosis) and positions LMWE as complementary to existing herbal products for xerostomia while offering Brassicaceae-specific mechanisms that merit further clinical testing.

## 5. Conclusions

This study provides quantitative evidence that LMWE protects against radiation-induced salivary gland dysfunction. In C57BL/6J mice, LMWE treatment reduced secretion lag time from 197.8 ± 9.2 s in the radiation-only group to 163.8 ± 6.5 s and 159.4 ± 6.1 s in the 6 and 12 mg/kg BW groups, respectively. LMWE also restored gland weight close to control values and enhanced amylase activity by over 20% at 12 mg/kg BW. Histological analysis revealed preserved acinar structure, reduced collagen I deposition, and alleviated fibrosis, while immunofluorescence showed recovery of Mist1, AQP5, and amylase expression. In vitro, LMWE attenuated TGF-β1–induced senescence, rescued mitochondrial respiration, and maintained membrane potential. In 3D salivary gland organoids, LMWE, and its active components DHPPD and E4Z-PD, selectively promoted acinar and ductal differentiation, respectively, supporting lineage-specific regeneration. Together, these findings highlight LMWE as a promising, low-toxicity therapeutic candidate for xerostomia, offering both functional preservation and regenerative potential through antioxidant, anti-senescent, and differentiation-enhancing effects.

## 6. Limitation

Present study demonstrated that LMWE attenuated radiation-induced fibrosis and restored acinar function, we acknowledge that direct evidence for modulation of the TGF-β1 signaling cascade was not obtained. Nonetheless, our findings including reduced collagen I accumulation, alleviation of acinar atrophy, and recovery of functional markers such as AQP5 and amylase are consistent with a suppression of TGF-β1 activity, given that this pathway is a well-established driver of salivary gland fibrosis and secretory dysfunction after irradiation [39]. Moreover, previous reports have shown that bioactive components of Lepidium meyenii, such as macamides and glucosinolates, exert anti-fibrotic and antioxidant effects in part through the modulation of TGF-β1/Smad signaling or related pathways [38]. These indirect lines of evidence support our mechanistic interpretation. Future studies incorporating molecular analyses, such as Western blotting for Smad phosphorylation, immunofluorescence, or transcriptomic profiling, will be required to definitively establish LMWE’s role in regulating the TGF-β1 axis. Although these results are promising, several limitations should be addressed in future studies. However, the long-term safety, pharmacokinetics, and systemic bioavailability of LMWE and its active metabolites remain unclear. Additionally, the efficacy of human-derived salivary gland models has yet to be tested. Further studies incorporating lineage tracing and single-cell transcriptomics may elucidate the precise mechanism of action of LMWE and its role in tissue homeostasis. Combining LMWE with biocompatible delivery platforms, such as injectable hydrogels or scaffold-based organoid constructs, may also improve its clinical applicability and therapeutic precision.

## Figures and Tables

**Table 1:** The primer sequence for real-time RCR.

Gene	Forward Primer	Reverse Primer
Aqp5	5’-ATT GGC TTG TCT GTC ACA CTG G -3’	5’-AAT AGG CCC TAC CCA GAA GAC -3’
Amy1	5’-TCT GGG TGG TGA AGC AGT GT-3’	5’-AAG GGC TCT GTC AGA AGG CA -3’
Sox9	5’-ATC TTC AAG GCG CTG CAA GCG -3’	5’-ACT TTC CCA GCT TGC ACG TCT G -3’
Krt14	5’-CAT CGT CTC AAT TCT CCT CTG GC -3’	5’-AAG CCT GAG CAG CAT CTA GA G -3’
Krt18	5’-GAA CAT CAA GGT CAA GCT TGA G -3’	5’-GAA CTC TGG TAT CAT TGG TCT C -3’
Charm1	5’-CTT CAT CCT CAC CTG GAC ACC -3’	5’-GTT GCA CAG TGC ATA GCA CAT GG -3’
Charm3	5’-GCT GTG CTA TAT CAA CAG CAC CG -3’	5’-ACC GAC TGT CTC TGC TGG TAC -3’
GAPDH	5’-TGC CCC CTC TGC TGA TGC C-3’	5’-CCT CCG ACG CCT GCT TCA CCA C-3’

Aqp5, aquaporin 5; Amy1, amylase alpha 1; Sox9, SRY-box transcription factor 9; Krt14, keratin 14; Acta2, actin alpha 2; GAPDH, glyceraldehyde 3-phosphate dehydrogenase.

**Table 2:** Comparison of biochemical parameters in experimental C57BL/6 mice.

	Sham Control			Radiation Injury			Radiation Injury LMWE 6mg/kg BW			Radiation Injury LMWE 12mg/kg BW		
	Baseline *n* = 10	6 Weeks *n* = 10	12 Weeks *n* = 5	Baseline *n* = 10	6 Weeks *n* = 10	12 Weeks *n* = 5	Baseline *n* = 10	6 Weeks *n* = 10	12 Weeks *n* = 5	Baseline *n* = 10	6 Weeks *n* = 10	12 Weeks *n* = 5
**BW (g)**	18.8 ± 1.0	22.0 ± 0.9	25.1 ± 0.9	18.7 ± 1.8	21.9 ± 0.9	25.5 ± 0.8	18.4 ± 0.6	21.8 ± 1.1	25.6 ± 0.7	18.2 ± 1.1	21.9 ± 0.8	25.8 ± 0.3
**BUN (mg/dL)**	25.5 ± 1.5	27.6 ± 1.4	27.6 ± 2.3	24.4 ± 1.8	26.8 ± 1.6	27.6 ± 1.6	25.9 ± 2.1	29.4 ± 2.5	26.7 ± 2.0	27.5 ± 1.8	26.7 ± 1.9	25.7 ± 1.5
**Cr (mg/dL)**	0.5 ± 0.2	0.6 ± 0.1	0.7 ± 0.2	0.5 ± 0.2	0.7 ± 0.2	0.7 ± 0.2	0.6 ± 0.2	0.4 ± 0.1	0.7 ± 0.2	0.5 ± 0.3	0.6 ± 0.2	0.5 ± 0.1
**ALT (IU/L)**	29.3 ± 1.9	29.7 ± 3.8	28.0 ± 2.8	27.5 ± 1.3	30.5 ± 2.4	25.8 ± 1.7	29.8 ± 2.9	29.9 ± 2.7	31.7.0 ± 2.6	30.1 ± 1.9	29.8 ± 2.0	29.4 ± 1.9
**AST (IU/L)**	38.5 ± 1.3	39.5 ± 3.7	39.8 ± 4.3	35.7 ± 3.8	36.2 ± 5.4	37.6 ± 3.4	36.2 ± 4.2	37.3 ± 5.5	36.6 ± 4.3	36.2 ± 5.5	32.9 ± 5.8	38.5 ± 2.7
**LDH (IU/L)**	86.6 ± 27.9	88.9 ± 35.7	92.7 ± 19.7	90.8 ± 27.7	198.4 ± 62.7	96.2 ± 31.5	93.2 ± 32.6	123.5 ± 56.1	99.7 ± 42.0	87.1 ± 29.5	135.7 ± 43.1	93.7 ± 37.2
**CRP (mg/dL)**	30.3 ± 0.9	28.9 ± 6.7	33.7 ± 9.5	39.2 ± 9.5	39.8 ± 5.7	27.5 ± 10.5	22.7 ± 9.9	34.6 ± 16.7	36.7 ± 8.9	32.5 ± 8.5	42.5 ± 9.7	37.0 ± 9.9

LMWE, *Lepidium meyenii* Walpers extraction; BW, body weight; Cr, creatinine; BUN, blood urea nitrogen; ALT, alanine transaminase; AST, aspartate transaminase; LDH, lactate dehydrogenase; CRP, C reactive protein.

## Data Availability

The data presented in this study are available on request from the corresponding author due to privacy.

## Abbreviations

ALT	Alanine aminotransferase
AST	Aspartate aminotransferase
BUN	Blood urea nitrogen
CRP	C-reactive protein
DHPPD	(1*E*,4*Z*)-1-(2,4-dihydroxyphenyl)-5-(3,4-dihydroxyphenyl) penta-1,4-dien-3-one
DMEM	Dulbecco’s Modified Eagle Medium
DMSO	Dimethyl sulfoxide
E4Z-PD	(*Z*)-*N*-phenyldodec-2-enamide
EGF	Epidermal growth factor
FBS	Fetal bovine serum
FGF-2	Fibroblast growth factor 2
H&E	Hematoxylin and eosin
IHC	Immunohistochemistry
IF	Immunofluorescence
ITS-G	Insulin–transferrin–selenium supplement
JC-1	5,5′,6,6′-Tetrachloro-1,1′,3,3′-tetraethylbenzimidazolylcarbocyanine iodide
LDH	Lactate dehydrogenase
LMWE	Lepidium meyenii extract
OCR	Oxygen consumption rate
Par-C10	Rat parotid acinar cell line
PAS	Periodic acid–Schiff
RT-qPCR	Reverse transcription quantitative polymerase chain reaction
TGF-β1	Transforming growth factor beta 1
TEER	Transepithelial electrical resistance
